# Investigating the Effect of Presentation Mode on Cognitive Load in English–Chinese Distance Simultaneous Interpreting: An Eye-Tracking Study

**DOI:** 10.3390/jemr18060073

**Published:** 2025-12-01

**Authors:** Xuelian (Rachel) Zhu

**Affiliations:** National Institute of Education, Nanyang Technological University, Singapore 637616, Singapore; xuelian.zhu@nie.edu.sg

**Keywords:** technology-mediated interpreting, cognitive load, distance simultaneous interpreting, eye-tracking, performance scores, presentation mode

## Abstract

Distance simultaneous interpreting is a typical example of technology-mediated interpreting, bridging participants (i.e., interpreters, audience, and speakers) in various events and conferences. This study explores how presentation mode affects cognitive load in DSI, utilizing eye-tracking sensor technology. A controlled experiment was conducted involving 36 participants, comprising 19 professional interpreters and 17 student interpreters, to assess the effects of presentation mode on their cognitive load during English-to-Chinese DSI. A Tobii Pro X3-120 screen-based eye tracker was used to collect eye-tracking data as the participants sequentially performed a DSI task involving four distinct presentation modes: the Speaker, Slides, Split, and Corner modes. The findings, derived from the integration of eye-tracking data and interpreting performance scores, indicate that both presentation mode and experience level significantly influence interpreters’ cognitive load. Notably, student interpreters demonstrated longer fixation durations in the Slides mode, indicating a reliance on visual aids for DSI. These results have implications for language learning, suggesting that the integration of visual supports can aid in the acquisition and performance of interpreting skills, particularly for less experienced interpreters. This study contributes to our understanding of the interplay between technology, cognitive load, and language learning in the context of DSI.

## 1. Introduction

Distance simultaneous interpreting (DSI) is a technology-mediated interpreting mode wherein interpreters work remotely and are provided with audio or audiovisual feeds of speakers from different locations to deliver interpreting service in real time [1,2]. A widely acknowledged challenge in the general field of interpreting, DSI included, concerns the impact of cognitive load on the performance of interpreters [3,4,5,6,7,8,9]. In DSI, cognitive load is defined as the mental workload imposed on the interpreters when they are concurrently listening to and watching the video presentation while interpreting the input in one language into another [2,10].

Research shows that the video presentation mode, which dictates how auditory and visual inputs of the source speech are delivered to interpreters, plays a crucial role in influencing the cognitive load experienced during DSI [11,12,13,14,15,16]. For example, Napier et al. [12] found that video-based interpreting increased visual monitoring demands compared with audio-only modes. Seeber [13,14] demonstrated that simultaneous exposure to slides and speakers induces split-attention effects, heightening cognitive load when visual and auditory information are spatially separated. Conversely, Bertozzi and Cecchi [11] and Yuan and Wang [16] observed that well-aligned visual cues can ease working-memory demands by supporting message anticipation. Collectively, these studies indicate that cognitive load varies depending on how presentation modes coordinate visual and auditory input. This presentation can vary through interface designs, including visuals paired with audio, visuals alongside audio and text, or terminologies and numbers via software, each with different implications for the cognitive load of the interpreters [12,16,17,18]. It is important to examine which types of visuals are made available to interpreters during DSI, as these externally provided inputs can influence their cognitive processing and performance [9,10,19,20,21,22,23]. This line of inquiry underscores the critical role of carefully selecting visual content in DSI to manage the cognitive load effectively [24,25,26].

In addition, research indicates that interpreter-centric factors play a significant role in determining interpreters’ cognitive load, with interpreters’ experience arguably being the primary determinant of their performance and cognitive load [3,27,28,29]. In this study, experience is distinctly defined in terms of interpreters’ familiarity and proficiency in performing (distant) simultaneous interpreting [30,31,32,33,34,35,36,37]. A plethora of studies have attempted to explore the differences in cognitive load between different levels of interpreters [38,39,40,41,42], but it is not clear whether the existing differences could be replicated in the distance working mode, and whether the effect of experience level might interact with the presentation mode discussed earlier.

Against such a backdrop, this study aims to investigate how the presentation mode of video input affects the cognitive load of professional and student interpreters in English-Chinese DSI. Specifically, eye-tracking has been widely recognized as a reliable method for examining real-time cognitive processing and attentional allocation [43,44,45], providing the methodological foundation for the present study. The eye-tracking measures, when combined with proficiency assessments, provide a more thorough gauge of interpreter performance and cognitive load [46,47,48]. This multifaceted approach, framed within a rigorously structured research design, helps demystify the complex interplay of physiological, technological, and cognitive factors influencing interpreters in DSI.

Two research questions (RQs) guide the study:

RQ1: How do different presentation modes influence the cognitive load of professional and student interpreters in English–Chinese DSI as measured by eye-tracking measures and performance scores? (a within-group comparison).

RQ2: What are the differences in cognitive load between professional and student interpreters under four presentation modes in English–Chinese DSI? (a between-group comparison).

## 2. Literature

### 2.1. Cognitive Load in DSI

Cognitive load in DSI is defined as a multifaceted construct that reflects the mental load placed on an individual’s cognitive system while executing a specific task [49,50,51,52]. In the field of interpreting studies, there have been multiple theoretical frameworks intended to define cognitive load. One of the most well-known frameworks is Gile’s Effort Model, which defines cognitive load in terms of listening effort, decision-making effort, and production effort [53,54]. Another influential framework is Seeber’s cognitive load model, which conceptualizes cognitive load in terms of auditory processing load, linguistic processing load, memory load, and decision-making load [14,55]. This complexity in defining cognitive load suggests that there is no single or perfect way to operationalize and measure this construct. In this study, cognitive load in DSI is operationalized as mental load—the cognitive demand of the DSI tasks and environment—and mental effort—the total work done by the interpreter to complete the task [56]. That is, task and environment factors impose mental load on the interpreter, who then devotes measurable mental efforts to perform the task [3,52,57].

The task and environment factors thus comprise task-specific factors, linguistic and paralinguistic features of the input, as well as environmental features. The interpreter factors, on the other hand, encompass a set of cognitive, affective, and experiential resources that interpreters draw upon during task performance. These include linguistic knowledge (e.g., lexical and syntactic proficiency in both working languages), topic knowledge (familiarity with the subject matter), personal traits (such as stress tolerance, attentional control, or cognitive flexibility), technology awareness (the ability to navigate and manage digital platforms used in DSI), interpreting strategies (e.g., chunking, anticipation, reformulation), and (meta)cognitive processes (such as planning, monitoring, and evaluating one’s own performance). These factors shape how interpreters allocate mental resources, influencing both their cognitive load and overall performance. It is suggested that the completion of an interpreting task is the result of the interaction between task/environment factors and interpreter factors [58].

One of the recognized issues in DSI is the role of these factors in affecting cognitive load and performance [7,8,9]. Understanding these influences, particularly the mode-specific nature of DSI, is key to determining whether the distance mode of working has a positive or adverse effect on interpreters’ cognitive load.

### 2.2. Presentation Mode of Video Input in DSI

Interpreters, whether in the booth or working remotely, encounter various modes of video or visual input such as slides, the speaker, the audience, or the speaker’s draft [5,11,59,60]. The presentation mode of video input refers to what is presented on the screen—such as video clips of speakers, audience, slides, or conference halls [2,12]. Currently, DSI functions have been incorporated into various video-conferencing platforms, including but not limited to Google Meet, Microsoft Teams, Webex, and Zoom [61]. Additionally, a multitude of independent DSI-specific platforms, such as Interprefy, KUDO, and VoiceBoxer, as well as virtual booth solutions like Ablio, Cymo, and GreenTerp, have emerged and flourished [62].

Although these platforms differ in design, a common feature is that they typically allow interpreters some degree of agency in customizing their visual workspace—for example, displaying slides and the speaker side by side, positioning the speaker in a corner, or adjusting window sizes. However, this flexibility is often limited by platform defaults and interface constraints, meaning that interpreters cannot fully control what visual elements are available but can only reorganize the given components. This partial but meaningful degree of customization highlights why understanding visual presentation modes is crucial: while experienced interpreters may intuitively configure their workspace effectively, novice interpreters may lack the expertise to make optimal choices. Therefore, empirical research is needed to determine which presentation configurations support or hinder cognitive processing, providing evidence-based guidance for interpreter training.

Four specific modes of input presentation—reflecting the most common configurations used in DSI—were chosen for investigation in the present study. These modes consist of the speaker video input mode [5,61], PowerPoint slides mode [40,41,63], a split mode showing the speaker and slides side by side [13], and a corner mode where slides occupy most of the screen with the speaker in a corner [11,59].

#### 2.2.1. Speaker Mode

Speaker mode, which features the presence of the speaker’s face and body, is incorporated because nonverbal communication is a crucial aspect of interpreter-mediated communication [64]. Nonverbal cues, such as gestures, facial expressions, and postures (kinesics), provide valuable embedded messages beyond spoken words [65]. Among these, facial clues are perhaps the most crucial carriers and have been referred to as “effect displays”, reflecting a universal aspect of human communication [66]. Jesse et al. [60] and Gieshoff [5] used similar designs to investigate the impact of lip movement on interpreting performance by providing two sources of information: the auditory speech and accompanying lip movements, as opposed to presenting only the auditory speech. In this study, videos of the speaker’s speech delivery, showing the speaker’s upper body, were selected for presentation to the interpreters.

#### 2.2.2. Slides Mode

With the rising prevalence of PowerPoint slides in various real-life conference settings, research in SI has begun to incorporate slides as the instrument and consider the influence of multimodal input rising with its popular use [40,41,63,67,68,69]. Körmendy [63] and Kroesen [69] studied the effect of PowerPoint slides on SI interpreters, finding that their presence can increase cognitive load, especially for less experienced interpreters. Both studies highlight limitations due to small sample sizes and participants’ lack of real-life SI experience, yet underline the significant impact of visual aids on SI performance. Building on this work, Seeber [13], Korpal and Stachowiak-Szymczak [40,41], and Stachowiak-Szymczak and Korpal [68] used eye-tracking to more precisely examine how interpreters coordinate visual attention when slides are present. These studies consistently showed that slides draw significant visual focus and can alter gaze patterns, often increasing fixation duration and saccade frequency. Such findings indicate that visual materials can meaningfully reshape interpreters’ cognitive processing pathways.

#### 2.2.3. Split Mode

Building upon the preceding discussions highlighting the significance of nonverbal elements and the role of slides in DSI, it is compelling to bring these two factors together for a comprehensive comparison. Seeber [13] made an initial attempt to use eye-tracking to capture the SI interpreters’ online fixations on numbers under visual modality (with the speaker’s face, gestures, or slides) and auditory modality. Seeber’s [13] eye-tracking research is contrasted by the findings of Kamiya [70]. While Seeber’s study revealed that interpreters often focus on the speaker’s face for cues aiding in verbal processing or as an early behavior mechanism, Kamiya’s [70] listening research presents a different perspective. In this later study, participants, irrespective of their experience levels, showed a general preference for observing the whole body of the speaker.

#### 2.2.4. Corner Mode

More recently, the major video-conferencing platforms, particularly Zoom, have started to gain traction, resulting in the “Zoom boom” in interpreting [60]. In their survey of 311 professional interpreters, Chmiel and Spinolo [60] found that 78.5% of respondents used Zoom even though it is not the only platform for DSI. Bertozzi and Cecchi [61] suggested that the best practice of visual presentation is to switch to a side-by-side view to see the slides and the speaker simultaneously, with a survey study. Saeed et al. [10] conducted an investigation on professional interpreters to explore their preferences over distance interpreting interface design, finding that the interpreters prefer minimalist designs like Zoom, as opposed to the interfaces that feature complex information and options. The default configuration in Zoom positions the speaker in the right corner, while simultaneously projecting visual materials like slides onto a larger screen. This setup aligns with the design of the Corner mode as Slides Primary and Speaker at Corner outlined in the present study.

Taken together, these four presentation modes represent the most commonly used configurations in contemporary DSI practice, providing a clear foundation for the experimental design described in the next section.

## 3. Methodology

### 3.1. Motivation and Aims of the Study

This study was conducted to investigate how different presentation modes of video input affect cognitive load in distance simultaneous interpreting (DSI), with a focus on differences between professional and student interpreters. The methodology is therefore organized to address the two research questions concerning within-group and between-group comparisons.

The overall research design was a between- and within-subject design with two factors: the presentation mode of video input (the Speaker, Slides, Split, and Corner modes) and experience level (professional group and student group). In the eye-tracking data collection procedure, the entire screen of each presentation mode was designated as the area of interest (AOI).

### 3.2. Study Context and Timeline

The experiment was conducted between April 2021 to March 2022 in Singapore and China amid the global pandemic.

At the time of the experiment, the professional interpreters had gotten used to working remotely, while the students had attended online interpreting classes, indicating that both groups had experience in receiving video presentations via a computer screen while interpreting.

### 3.3. Participants

Among the 36 participants in the main study, 19 were professional interpreters, while 17 were student interpreters. 28 were females, and 10 were males. All professional interpreters had served over 200 international conferences, and while all held professional certificates, student interpreters were from the Master of Translation and Interpreting (MTI) programs from a tertiary institution.

### 3.4. Ethical Considerations

The study received ethical clearance from the Institutional Review Board (IRB) at the researcher’s university. Participation in this study was entirely voluntary. Each participant received SGD 40 as compensation for their time.

### 3.5. Stimuli and Materials

#### 3.5.1. Choice of Stimulus

The DSI tasks, used as the instruments, were scheduled to be presented to the participants consecutively for the four presentation modes. The National Day Message 2019 delivered by Prime Minister Lee Hsien Loong was chosen (see Appendix A for the transcripts). Seven minutes out of the 8 min and 56 s video were chosen and later divided into four clips with approximately the same duration, although they differ slightly because each clip was cut off at the end of a sentence, avoiding sentence chunks in the middle. The lengths of these four sections are 112 s, 103 s, 95 s, and 110 s, with speech rates of 107 w/min, 121 w/min, 114 w/min, and 124 w/min, respectively. A one-sample *t*-test was conducted to compare the speeds of the four models with the accepted speech rate of 130 words/minute (w/m), which is recommended by AIIC [71]. Coh-Metrix [72] was used to compare and confirm the similarity of the linguistic content of the four segments, the syntactic and lexical parameters, as well as the information density, of each of the four clips of segmentation.

#### 3.5.2. Slide Design

Based on the structure of the speech, the type of phrases, and the information intensity [73,74], PowerPoint slides in this study were designed to present one topic sentence and four key phrases on a background color of light grey. All used the Calibri font, 1.5 space, but differed in font size: 44 for the topic sentence phrase, 28 for the supporting points phrase. Margins of 2 cm were inserted around the edge of the screen to reduce track loss.

### 3.6. Apparatus and Procedure

#### 3.6.1. Apparatus

The stimuli were presented on an LCD screen with 1920 × 1080 pixel resolution. Participants’ eye movements were recorded using a Tobii Pro X3-120 screen-based eye tracker (Danderyd, Sweden) [75] with a sampling frequency of 120 Hz. Tobii Pro X3-120 has been applied in various recent language studies and proved useful and reliable [76,77]. The computer software Tobii Pro Lab 1.162 [75] was installed on the password-protected laptop of the investigator for data recording and data processing. A calibration procedure for eye-tracking was conducted prior to the interpreting session. Four types of eye-tracking measures were collected and used to answer the research questions of the study: total fixation duration (TFD), the total time spending on an area of interest (AOI); fixation duration (FC), the number of fixations in an AOI; total visit duration (TVD), the total time spending on AOI across visits, including both the fixations and the saccades; and visit count (VC), the number of visits in an AOI. These measures were found to correlate with cognitive load in interpreting studies, with higher durations or counts indicating higher cognitive load, and vice versa [26,48,78].

#### 3.6.2. Procedures

Participants were tested individually in a university computer laboratory, where they underwent eye-tracking calibration, performed the English–Chinese DSI tasks under the four presentation modes, and completed the measures used to assess cognitive load and interpreting performance. They were asked to read the instructions and sign an informed consent form with the IRB approval details. They were verbally informed that the interpreting test was a simulation of DSI and that participants would be expected to listen to a speech through earphones while watching the screen, on which the speaker and/or slides would be presented. They were noted that the DSI test consists of four sections, each accompanied by a specific presentation mode. Participants were directed to take a comfortable seated position in front of the eye tracker and were provided with earphones equipped with a speaker to enable the recording of their interpreting. Following that, participants were instructed to keep their eyes on the screen and start the interpreting session to facilitate the collection of gaze behavior.

After collecting the data, a rating procedure was conducted by four raters who are experienced interpreting instructors based on a rating scale proposed by Han (2015, 2017; Appendix B Table A1) [79,80]. The four raters participated only in the performance evaluation process and were not involved in the study design, data collection, analysis, or authorship. The results were verified and validated by the many-facet Rasch model (MFRM, [81]).

### 3.7. Data Analysis

A generalized estimating equation (GEE) model was applied to analyze the performance data. A GEE model can effectively capture the correlation of responses within subjects across multiple testing instances, thus accounting for dependency among the data [82,83,84]. GEE’s provision of consistent population-averaged effect estimates, regardless of the within-subject correlation structure, presents an advantage over traditional ANOVA methods that might inflate type I error rates due to the assumption of observation independence [85].

This study followed the guidelines provided by Bates et al. (2015a, b) [86,87] for conducting a series of linear mixed effect models (LMEMs) to analyze the gaze behavior data using the lme4 package [86] and lmerTest [88] in RStudio, Version 2023.9.1+494 [89] to answer the research questions of the study. Following Bates et al. (2015a) [86], the minimal-to-maximal approach was adopted, estimating the t and *p* values of each fixed and random variable to determine which one reached statistical significance (*p* < 0.05). The LMEM analysis and its potential application in interpreting studies were reported in a separate manuscript. For selected TFD, FC, and VC models, there were three fixed effects—presentation mode, experience level, and their interaction—while the model also accounted for the random slope of presentation mode nested within participants. For the selected TVD model, the random slope of presentation mode was also taken into account. After running the models, the post hoc analysis conducted using the “emmeans” package in R yielded the average values for each level of the predictor variables, namely the presentation mode and the experience level.

### 3.8. Visual Representation

**Figure 1:**
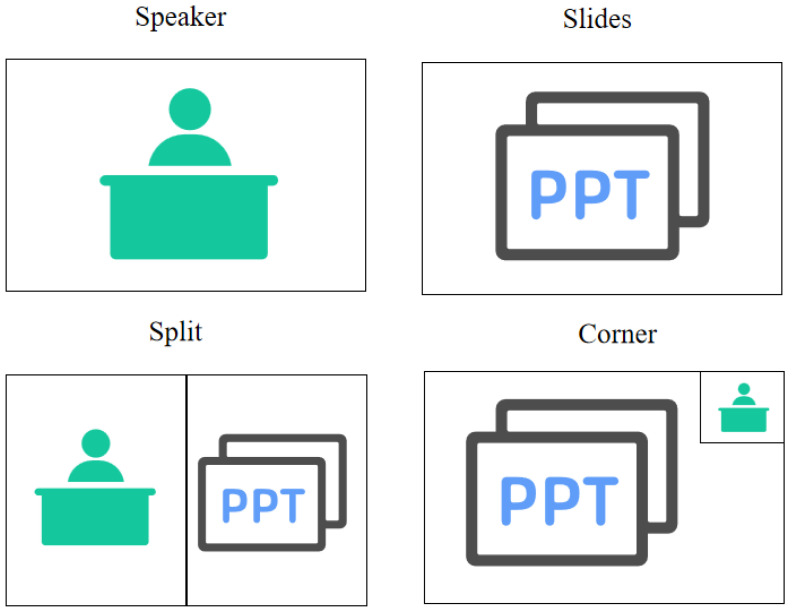
Visual representation of the four presentation modes.

A visual representation of the four modes is displayed in Figure 1.

## 4. Results

### 4.1. Performance Scores

**Figure 2:**
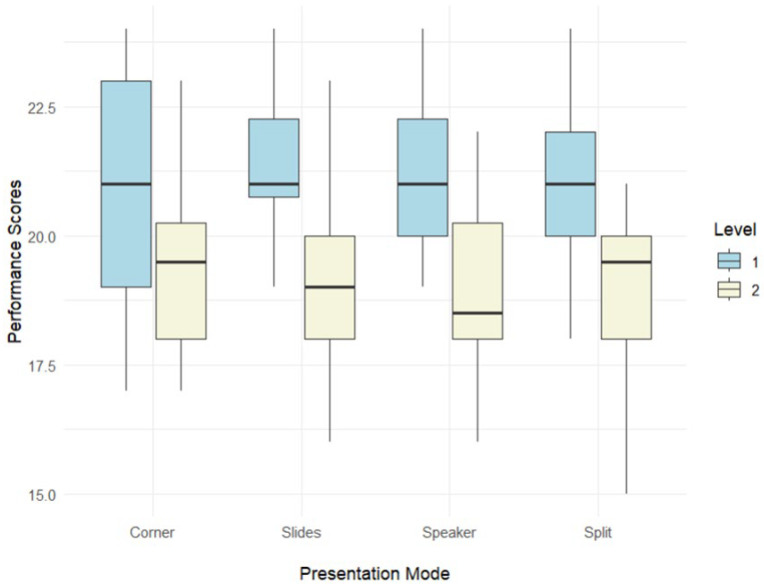
The differences in performance scores between the professional and student groups. Note. Level 1: professional group; Level 2: student group.

The professional group had comparatively higher performance scores than the student group across four presentation modes, as indicated in Figure 2. Descriptive statistics of the performance scores in each presentation mode, including the mean, standard deviation, skewness, and kurtosis, are presented in Appendix C Table A2. Specifically, the professional group achieved higher scores in Speaker mode (M = 21.5; SD = 1.67) compared to the student group (M = 19.0; SD = 1.80). This pattern was also observed in other presentation modes: In Slides mode, the professional group similarly had higher scores (M = 21.5; SD = 1.54) compared to the student group (M = 16.4; SD = 2.00); in Split mode, the professional group (M = 21.1; SD = 1.76) again outperformed the student group (M = 19.1; SD = 1.56); and finally, in the Corner mode, the professional group had higher scores (M = 21.3; SD = 1.86) compared to the student group (M = 19.3; SD = 1.83). In addition, the skewness and kurtosis indices indicate a normal distribution since they fell within the above-mentioned range [90].

The findings from generalized estimating equation (GEE) analysis indicate that the professional group achieved a significantly higher mean score than the student group (2.724, SE = 0.584, *p* < 0.0001). In addition, in each presentation mode, the professional group consistently outperformed the student group. Furthermore, regardless of the experience level of the participants, the overall scores in Corner mode were significantly lower than those in Speaker mode (0.884, SE = 0.270, *p* = 0.0006), the Slides mode (0.609, SE = 0.150, *p* < 0.0001), and the Split mode (0.678, SE = 0.170, *p* < 0.0001). Notably, within the professional group, scores in Corner mode were lower than those in Speaker (0.628, SE = 0.154, *p* = 0.004) and Split (0.878, SE = 0.232, *p* = 0.004). Similarly, the student group exhibited lower scores in Corner mode compared to Speaker mode (1.352, SE = 0.388, *p* = 0.014).

### 4.2. Eye-Tracking Measures

The results of pairwise comparisons of the eye-tracking measures, with Bonferroni corrections to control for the effect of multiple comparisons, are presented in this section.

The significant results of the TFD post hoc analysis are presented in Table 1. The table shows that the professional group had significantly longer TFD in the Slides mode than that in the Split mode (27.1 s) and the Corner mode (22.82 s), while the student group had significantly longer TFD in the Slides mode than that in the Speaker mode (39.05 s), the Split mode (50.15 s) and the Corner mode (55.32 s), respectively. Moreover, the TFD of the student group was higher than that of the professional group across four presentation modes, but they did not reach statistical significance.

The significant results of the FC post hoc analysis are presented in Table 2. As demonstrated in the table, in examining the FC across various presentation modes and experience levels, a clear pattern emerges. The Slides mode is associated with significantly higher FC compared to the Speaker, Split, and Corner modes. When considering experience levels, professionals exhibited a higher FC in the Slides mode over the Split mode (117.16) and a moderately higher FC in the Corner mode (91.11). Students displayed a lower FC in the Slides mode when contrasted with the Speaker (197.81), Split (225.62), and Corner modes (209.56).

Table 3 of pairwise comparisons reveals several trends in TVD with Bonferroni corrections applied. Firstly, the Slides mode consistently shows higher TVD when compared to the Speaker, Split, and Corner modes, with all comparisons yielding statistically significant results (*p* < 0.0001). Specifically, the Slides mode has a longer TVD than the Speaker mode and surpasses the Split and Corner modes by 78.22 and 68.53 units, respectively. In the context of professional experience, Slides mode has shorter TVD than the Speaker mode but longer TVD when compared to the Split and Corner modes, with the differences being statistically significant. On the other hand, in the context of student interpreters, the Slides mode has a longer TVD compared to both the Split and Corner modes, with substantial increases of 92.436 and 82.736 units, respectively, again with high statistical significance. The negative estimates for comparisons where the Speaker mode is less than the Slides mode suggest that the Speaker mode is associated with significantly shorter TVDs.

The significant results of the post hoc analysis with Bonferroni corrections applied for multiple comparisons are presented in Table 4. The Speaker mode has a significantly lower VC than the Slides mode (−5.490), while the Slides mode has a higher VC than the Split mode (5.065).

## 5. Discussion

This study aimed to examine the impact of presentation mode on cognitive load in English-to-Chinese DSI, assessed by performance score and gaze behaviors. We now provide the following discussions based on the results.

### 5.1. RQ1: The Within-Group Comparison of Performance Score and Gaze Behavior

The results showed that the professional group’s performance remained stable across four presentation modes, suggesting a resilient and flexible skill set in accomplishing the tasks regardless of the variations in visual aids, which is in line with previous research [40,41,68]. The stability in performance score seems to indicate that the training and experience the professional interpreters received prevented any possible influence of the presentation mode.

Despite the lack of any observable difference in their output, the gaze behavior of the professionals was affected by the presentation mode of the video input. It appears that the professional group experienced significantly lower cognitive load during Split mode, but the highest cognitive load in the Slides mode in comparison to the other presentation modes. In other words, when only the slide is displayed (the Slides mode), there is a noticeable increase in gaze duration and fixation count compared to when just the speaker’s face is shown (the Speaker mode) or when the face and slides are presented side by side (the Split mode) or when the speaker is placed in the corner window (the Corner mode). This indicates that slides containing key information, when viewed in isolation, might invite more active attention from the professional interpreter, thus inducing a greater cognitive load in this scenario [40,91]. Additionally, the fact that the study revealed variations in cognitive load among interpreters across different modes shows that some existing frameworks, such as Gile’s Effort Model of SI [54] (p. 167), might not be able to explain the complexities in DSI since they only consider the audio input as the only input. This is in line with Seeber’s [14,55,56] suggestion for considering visual input in SI research, especially given that visual elements are inherent components in DSI.

In contrast to the professional group, the student group displayed a notable trend in their performance across the presentation modes, with Slides mode showing the lowest performance level. However, it is important to note that this decrease in performance among the student group in Slides mode, while observable, did not achieve statistical significance when it was compared with the scores in other presentation modes. Overall, this outcome is consistent with the findings from previous research, such as Shao and Chai [92], which have identified a negative correlation between cognitive load and interpreting performance, particularly pronounced in novice interpreters. This suggests that higher cognitive demands in certain presentation modes may disproportionately impede the DSI performance of less experienced interpreters.

### 5.2. RQ2: The Between-Group Comparison of Performance Score and Gaze Behavior

The professional interpreters significantly outperformed student interpreters in all four presentation modes, which aligns with prior research indicating that professional interpreters typically exhibit superior performance as a result of their extensive training and practical experience in the field [40,41,93,94]. It is important to note that the four presentation modes in the DSI task were linguistically similar, with comparable lengths and delivery speeds of the source speech. This design ensures that the performance differences observed are more likely attributable to interpreter experience rather than variations in task characteristics. Furthermore, the context in which the data was collected, specifically during the pandemic period of 2021–2022, adds another layer of complexity. The two groups, professionals and students, have distinct experiences with DSI. The professional interpreters, having engaged in real-life DSI, brought a wealth of practical experience to the task. In contrast, the student group, primarily trained online during this period, may not have had a specific focus on DSI or SI in their curriculum. This difference in practical exposure to DSI contexts is likely a significant factor contributing to the consistently high performance of professionals across all presentation modes, in contrast to the relatively lower scores observed in the student group.

In addition, the eye-tracking data from this study provide revealing insights about cognitive load, as evidenced by the temporal and count measures of gaze. In Slides mode, student interpreters exhibited notably longer TFD than their professional counterparts, which indicates a higher cognitive load [40,41,48,91]. This longer duration suggests that students engage in extended visual text searches to aid their interpretation, implying a reliance on visual support for their output [40,41,91]. On the other hand, professional interpreters, possibly due to their experience, seem to have automatized the interpreting process. This automatization allows them to distribute their attention more effectively, without heavily depending on visual stimuli [27,28,40,42,95]. Another noteworthy observation in Slides mode, despite not reaching statistical significance, is the higher number of FCs for the student group compared to the professionals. This is suggestive that students faced additional cognitive challenges, possibly struggling to efficiently locate necessary information, leading to more frequent eye movements.

In the Split mode, a disparity between professional and student interpreters is that professional interpreters benefited from visual cues to reduce their cognitive load, as indicated by their TFD, while the student interpreters did not experience the same benefit. This observation aligns with Jesse et al.’s findings [62], where the participants were also student interpreters who did not benefit from visual cues to alleviate cognitive load. This discrepancy is likely due to inexperienced interpreters not yet having developed the skill to effectively process and synthesize multiple sources of information simultaneously. This distinction highlights that the usefulness of visual aids in interpreting is not universally advantageous but rather contingent on the interpreter’s level of expertise.

## 6. Limitations

The findings of this study should be interpreted with some limitations in mind, which, in turn, point to potential directions for future research in the field.

First, this study tapped into the presentation mode, the most prominent factor affecting the cognitive load in DSI, but it did not investigate other possible factors [57]. These factors, such as DSI platforms, the body language of speakers, participant interactivity, or interpreters’ technology awareness, are worth further investigation, given that the development of technology has brought about more variance in technology-mediated interpreting. Future researchers can further investigate the various factors influencing DSI.

Second, although the scoring categories and their thresholds were generally effective and performed well, a larger sample size would provide a more accurate estimation of various parameters in the MFRM analysis. That explains why I retained all available data in the analysis and employed robust methods to handle missing data, even though some participants had missing eye-tracking data. Although the sample size (N = 36) may appear modest, it exceeds that of most previous interpreting eye-tracking studies and provides a large number of observations through repeated measures across four presentation modes. Nonetheless, obtaining a large sample size is a consistent challenge in the field of DSI. The current study, in fact, is relatively large compared to many preceding studies that have applied sensor technologies in the contexts of DI and/or DSI.

Third, while this study is situated within the evolving landscape of technology-mediated interpreting, its focus and scope can be extended. As technology continues to advance rapidly, it has made significant inroads into the field of interpreting. Notably, technology-assisted tools, such as live captioning in DSI, terminology tools, or number projectors, have received widespread adaptation and popularity (e.g., [18,96,97,98,99]). Future research could investigate how different technological interfaces or visual aids in DSI can affect interpreter performance, thereby contributing to a more comprehensive understanding of cognitive load in this exciting field of human–machine interaction.

## 7. Conclusions

This study contributes to the intellectual understanding of the cognitive processes in DSI, shedding light on the nexus between cognitive psychology and interpreting research. By comparing the cognitive load of professional and student groups, this study provides insights for training and practice for DSI interpreters. Training programs can consider the findings of the current study in designing pedagogies to prepare trainee interpreters for the diverse challenges they may face in DSI. Specifically, the practices of professional interpreters can be leveraged as guiding principles in training as a strategic approach.

First, the study revealed that professionals exhibit lower cognitive load in the Split mode, which presents the slides and speaker video side by side, suggesting this presentation mode can be used to facilitate the management and processing of complex information. The findings resonate with Gile’s Effort Model in that interpreting performance depends on how well interpreters coordinate limited cognitive resources across listening, memory, and production efforts. While the Effort Model does not explicitly account for visual input, the Split mode may indirectly ease coordination and memory demands by providing a stable and predictable visual layout. This interpretation aligns more closely with Seeber’s multimodal framework, which conceptualizes interpreting as the integration of multiple sensory channels. Our results indicate that Split mode may optimize this cross-modal integration, thereby reducing cognitive strain and supporting improved performance. By analyzing how interpreters focus attention or prioritize information, training programs can develop techniques that emulate these successful practices.

Second, interpreter training programs may integrate techniques used by professional interpreters to manage cognitive load—such as prioritizing the speaker’s view even when slides are available—so that trainees can learn expert-like visual attention strategies through modelling [100,101]. At the same time, programs should equip trainees with the technological competencies needed for DSI by familiarizing them with common digital platforms and tools. Providing explicit guidance on setting up presentation software, managing visual inputs, and minimizing digital distractions helps ensure that technology functions as a facilitator rather than an additional cognitive burden, a skill set increasingly essential in today’s digitally mediated interpreting environment.

Third, given that the Split mode demonstrated the most balanced cognitive load and the highest interpreting performance among professionals, designers of distance interpreting platforms (e.g., Zoom, KUDO, Interprefy) may consider incorporating similar layout features to enhance user experience and interpreter efficiency. Streamlined interfaces that allow interpreters to access slides and speaker video in a stable and predictable configuration can help reduce unnecessary cognitive strain. Platforms may also explore offering customizable layouts that prioritize clear visual hierarchy and minimize digital distractions.

To summarize, this study enriches the academic discourse on cognitive aspects of interpreting and provides some insights for interpreter training programs, emphasizing the growing need for tech-savviness in the evolving landscape of the profession and language learning at large.

## Figures and Tables

**Table 1:** Pairwise comparison of the TFD Within the predictors.

Comparisons	Estimate	SE	df	t Ratio	*p* Value
Speaker < Slides	−23.193	5.29	97.8	−4.383	0.0002 ***
Speaker > Split	15.432	5.35	98	2.883	0.0246 *
Speaker > Corner	15.876	5.29	97.8	3	0.0177 *
Slides > Split	38.625	5.35	98	7.217	<0.0001 ***
Slides > Corner	39.069	5.29	97.8	7.383	<0.0001 ***
Professional Slides > Professional Split	27.10	7.16	97.8	3.787	0.0062 **
Professional Slides > Professional Corner	22.82	7.16	97.8	3.189	0.0388 *
Student Speaker < Student Slides	−39.05	7.80	97.8	−5.008	0.0001 ***
Student Slides > Student Split	50.15	7.96	98.2	6.300	<0.0001 ***
Student Slides > Student Corner	55.32	7.80	97.8	7.094	<0.0001 ***

Note. SE: standard error of measurement, * *p* < 0.05, ** *p* < 0.01, *** *p* < 0.001.

**Table 2:** Pairwise comparison of the FC within the predictors.

Comparisons	Estimate	SE	df	t	*p* Value
Speaker < Slides	−137.7	20.3	97.9	−6.768	<0.0001 ***
Slides > Split	169.314	23.8	34.1	7.128	<0.0001 ***
Slides > Corner	156.710	24.5	34.0	6.400	<0.0001 ***
Professional Slides > Professional Split	117.16	27.5	97.8	4.258	0.0012 **
Professional Slides > Professional Corner	91.11	27.5	97.9	3.311	0.0274 *
Student Speaker < Student Slides	−197.81	30	97.9	−6.597	<0.0001 ***
Student Slides > Student Split	225.62	30.6	98.3	7.37	<0.0001 ***
Student Slides > Student Corner	209.56	30	97.9	6.989	<0.0001 ***

Note. SE: Standard error of measurement, * *p* < 0.05, ** *p* < 0.01, *** *p* < 0.001.

**Table 3:** Pairwise comparison of the TVD within the predictors.

Comparisons	Estimate	SE	df	t	*p* Value
Speaker < Slides	−64.15	7.1	98.2	−9.04	<0.0001 ***
Slides > Split	78.22	7.17	98.6	10.905	<0.0001 ***
Slides > Corner	68.53	7.1	98.2	9.656	<0.0001 ***
Professional Speaker < Professional Slides	−45.806	9.6	98.2	−4.773	0.0002 ***
Professional Slides > Professional Split	64.004	9.6	98.2	6.669	<0.0001 ***
Professional Slides > Professional Corner	54.322	9.6	98.2	5.66	<0.0001 ***
Student Speaker < Student Slides	−82.498	10.5	98.2	−7.889	<0.0001 ***
Student Slides > Student Split	92.436	10.7	99	8.669	<0.0001 ***
Student Slides > Student Corner	82.736	10.5	98.2	7.911	<0.0001 ***

Note. SE: standard error of measurement, *** *p* < 0.001.

**Table 4:** Pairwise comparison of the VC within the predictors.

Comparisons	Estimate	SE	df	T Ratio	*p* Value
Speaker < Slides	−5.49	1.7	98.1	−3.227	0.0091 **
Slides > Split	5.065	1.72	98.3	2.944	0.0207 *

Note. SE: standard error of measurement, * *p* < 0.05, ** *p* < 0.01.

## Data Availability

The data supporting the findings of this study are not publicly available due to participant privacy and ethical restrictions in accordance with NTU-IRB approval (IRB-2019-07-022-01). De-identified data may be made available from the corresponding author upon reasonable request.

## Abbreviations

The following abbreviations are used in this manuscript:

DSI	Distance Simultaneous Interpreting
AIIC	International Association of Conference Interpreters
AOI	Area of Interest
TFD	Total Fixation Duration
FC	Fixation Count
TVD	Total Visit Duration
VC	Visit Count
MFRM	Many-Facet Rasch Model
GEE	Generalized Estimating Equation
LMEM	Linear Mixed-Effects Model
SI	Simultaneous Interpreting
MTI	Master of Translation and Interpreting

## Appendix A

### Appendix A.1. Transcripts of the DSI Tasks

#### Appendix A.1.1. Speaker Mode

These activities remind us that our history stretches back well before 1965, when we became independent, and even before 1819, when the British arrived. Singapore has drawn from many cultures and traditions in our journey towards nationhood. We have gone through many ups and downs.

This year, our economy has slowed down. Global demand and international trade have weakened. This has affected our manufacturing sector and trade-related services. In particular, we are feeling the worldwide cyclical downswing for electronics, which performed strongly last year. But other parts of our economy are still doing well. We have experienced such slowdowns before, and we will take this one in our stride. Should it become necessary to stimulate the economy, we will do so.

More fundamentally, the world is entering a more troubled period. We face grave challenges: one—economic uncertainties, with trade and globalisation under pressure; two—strategic risks, with growing frictions between the major powers; three—existential threats, with global warming and rising sea levels.

Singapore will not be immune to these global problems. On the economic front, they will disrupt supply chains, alter trade patterns, and shift investment flows. We must get ourselves ready for a very different future.

#### Appendix A.1.2. Slides Mode

But our past gives us confidence. Throughout our history, when trials and tribulations have beset us, we picked ourselves up and worked together to overcome them. Each time the world changed, we were able to survive. Each time, we re-invented and renewed our economy, our people, and our city, and we thrived again. This is what we must keep on doing.

For the economy, we are making good progress transforming our industries. We are servicing advanced jet turbines, researching new cures for diseases, and pushing boundaries in fintech services. Our seaport and airport are expanding to meet the growing demands of a dynamic Asia. The two integrated resorts, or IRs, are being enhanced to attract more tourists. Our tech and startup scenes are flourishing. Agencies like Enterprise Singapore are helping entrepreneurs and companies to strengthen, scale up, and expand into the international market.

We are also making good progress re-skilling and upgrading our workforce to be future-ready. SkillsFuture is building up the skills of tens of thousands of Singaporeans, helping them be more productive and employable, and preparing them for the new jobs being created. All these structural measures will not only address our longer-term challenges, but also help see us through a more immediate downturn.

#### Appendix A.1.3. Split Mode

By continuing to invest heavily in people, we enable each one of us to take advantage of new opportunities at every stage of life. This is a joint endeavour. The Government will keep on helping every citizen to achieve their potential and contribute their best to Singapore. Each one of us must strive to improve ourselves, do our best, and chase our dreams. And I know parents are making the effort to bring up children well, with the right character and values.

We intend to make preschool and tertiary education even more affordable, especially for lower and middle-income families. To help older Singaporeans, we have protected them for their healthcare and retirement needs. For those who wish to work longer, we will be raising the retirement and re-employment ages. I will say more about these matters at the National Day Rally.

Finally, we must continue to renew our city. Recently, a foreign leader, visiting us for the first time, told me that as his airplane flew over the island, he knew at once that he was over Singapore. Because, looking out of the window, he could see that every corner of the island had been meticulously thought through and lovingly tended—every housing precinct, every landmark, every patch of park and greenery.

#### Appendix A.1.4. Corner Mode

The island was a sparkling diamond, with brilliant facets catching the eye. Just like Jewel, at Changi Airport, where I am today. Many of you have already visited Jewel to explore its lush gardens, soak in the sight of the spectacular waterfall, and enjoy the unique ambience under the glass dome. Some of you may also work in the many new jobs created here. The Changi team conceived the concept of Jewel nine years ago, when Changi Airport was facing intense competition. Since its opening, Jewel has captured the imagination of both Singaporeans and visitors, and rightly so.

We are very proud of our new gateway to the world. It reminds us what makes this country special. It shows that Singaporeans not only have the creativity and daring to reinvent ourselves, but also the passion and the competence to turn dreams into reality. As you might expect, other cities and airports are already planning to emulate Jewel, and perhaps even do it bigger and better. But we dared to attempt the new, and we did it first.

Jewel is just one of many things we are doing to remake our city. Changi Terminal 5, Tuas Megaport, the Jurong Lake District, the redevelopment of Paya Lebar Airbase, and the Greater Southern Waterfront—all these projects will keep us busy and create new opportunities for Singaporeans for decades to come.

## Appendix B

**Table A1:** The rating categories and criteria for evaluating participants’ performance.

Subscales	Band 1 & Descriptors (Score Range 1–2)	Band 2 & Descriptors (Score Range 3–4)	Band 3 & Descriptors (Score Range 5–6)	Band 4 & Descriptors (Score Range 7–8)
Information Completeness	<40% of source text propositional content delivered in target text.	50–60% of source text propositional content delivered in target text.	70–80% of source text propositional content delivered in target text.	>90% of source text propositional content delivered in target text.
Delivery	Delivery lacks fluency. It is frequently hampered by disfluencies to such a degree that they may impede comprehension.	Delivery rather fluent. Acceptable, but with regular disfluencies.	Delivery on the whole generally fluent, containing a small number of disfluencies.	Delivery on the whole fluent, containing only a few instances of disfluencies.
Target Language Quality	Target language stilted and lacking in idiomaticity to such a degree that it may impede comprehension.	Target language is to a certain degree both idiomatic and correct. Acceptable, but contains many instances of unnatural and incorrect usage.	Target language generally idiomatic and, on the whole, mostly correct, with several instances of unnatural and incorrect usage.	Target language idiomatic and, on the whole, correct, with only a few instances of unnatural and incorrect usage.

## Appendix C

**Table A2:** Descriptive statistics of performance measures.

					Skewness		Kurtosis	
	Experience	N	Mean	SD	Skewness	SE	Kurtosis	SE
Speaker mode	Professional	19	21.5	1.65	0.05189	0.524	−0.87	1.01
	Student	17	19	1.8	0.14508	0.55	−0.519	1.06
Slides mode	Professional	19	21.5	1.54	0.19943	0.524	−0.616	1.01
	Student	17	18.9	1.76	0.3563	0.55	0.738	1.06
Split mode	Professional	19	21.1	1.58	−0.00157	0.524	−0.483	1.01
	Student	17	19.1	1.56	−0.89313	0.55	1.347	1.06
Corner mode	Professional	19	21.3	1.86	0.0636	0.524	−1.42	1.01
	Student	17	19.3	1.83	0.33957	0.55	−0.593	1.06
